# Clustering performance comparison using *K*-means and expectation maximization algorithms

**DOI:** 10.1080/13102818.2014.949045

**Published:** 2014-11-06

**Authors:** Yong Gyu Jung, Min Soo Kang, Jun Heo

**Affiliations:** ^a^ Department of Medical IT Marketing, Eulji University, Sungnam, Korea; ^b^ Department of Information and Communication, Kyungmin University, Seoul, Korea

**Keywords:** *K*-means, EM, logistic regression

## Abstract

Clustering is an important means of data mining based on separating data categories by similar features. Unlike the classification algorithm, clustering belongs to the unsupervised type of algorithms. Two representatives of the clustering algorithms are the *K*-means and the expectation maximization (EM) algorithm. Linear regression analysis was extended to the category-type dependent variable, while logistic regression was achieved using a linear combination of independent variables. To predict the possibility of occurrence of an event, a statistical approach is used. However, the classification of all data by means of logistic regression analysis cannot guarantee the accuracy of the results. In this paper, the logistic regression analysis is applied to EM clusters and the *K*-means clustering method for quality assessment of red wine, and a method is proposed for ensuring the accuracy of the classification results.

## Introduction

Clustering is an important means of data mining and of algorithms that separate data of similar nature. Unlike the classification algorithm, clustering belongs to the unsupervised type of algorithms. Two representatives of the clustering algorithms are the *K*-means algorithm and the expectation maximization (EM) algorithm. EM and *K*-means are similar in the sense that they allow model refining of an iterative process to find the best congestion. However, the *K*-means algorithm differs in the method used for calculating the Euclidean distance while calculating the distance between each of two data items; and EM uses statistical methods. The EM algorithm is often used to provide the functions more effectively.

Clustering means to split a large data set into a plurality of clusters of data, which share some trait of each subset. It is carried out by calculating the similarity or proximity based on the distance measurement method. The two can be divided into partial clustering and hierarchical clustering in the data. Hierarchical clustering can be agglomerative or divisive, i.e. bottom–up or top–down, respectively. It begins from each element and is intended to form a hierarchical cluster structure. The elements form a tree structure, which is a single cluster with all the elements on the other end. In this paper, the performance of both algorithms, EM and *K*-means, for quality assessment of red wine is compared by using logistic analysis. Experimental results are analysed and described by comparing two algorithms.

## Materials and methods

### Clustering algorithm overview

Clustering can be considered the most important unsupervised learning problem, and – like every other problem of this kind – it aims to find a structure (intrinsic grouping) in a collection of unlabelled data. A cluster is therefore a collection of objects which are ‘similar’ between each other and are ‘dissimilar’ to the objects belonging to other clusters (reviewed in [1]). Another kind of clustering is conceptual clustering in which two or more objects are considered to belong to the same cluster if it defines a concept common to all these objects. That is, objects are grouped according to their fit to descriptive concepts, not according to simple similarity measures.[2,3]

An important question is how to decide what constitutes good clustering, since it is commonly acknowledged that there is no absolute ‘best’ criterion which would be independent of the final aim of the clustering.[2,4] Consequently, it is the user who must supply the criterion that best suits their particular needs, and the result of the clustering algorithm can be interpreted in different ways. There are different types of clustering, which have been extensively reviewed.[2] Briefly, one approach is to group data in an exclusive way, so that if a certain item of data belongs to a definite cluster, then it could not be included in another cluster. Another approach, the so-called overlapping clustering, uses fuzzy sets to cluster data in such a way that each item of data may belong to two or more clusters with different degrees of membership. In this case, data will be associated to an appropriate membership value. Alternatively, in the third approach (hierarchical clustering), the algorithm begins by setting each item of data as a cluster and proceeds by uniting the two nearest clusters.[2] After a few iterations it reaches the final clusters wanted. Finally, the fourth kind of clustering uses a completely probabilistic approach. We examined the performance of two of the most used clustering algorithms: *K*-means and EM as follows.

### *K*-means clustering

The cluster analysis procedure is analysed to determine the properties of the data set and the target variable. It is typically used to determine how to measure similarity distance. Basically, it functions as follows:

Input: The number of *k* and a database containing *n* objects.
Output: A set of *k*-clusters that minimize the squared-error criterion.
Method:
  arbitrarily choose *k* objects as the initial cluster centres;
  repeat;
  (re)assign each object to the cluster to which the object is the most similar based on the mean value of the objects in the cluster;
  update the cluster mean, i.e. calculate the mean value of the object for each cluster;
  until no change.

To start using the clustering method, it can be divided into two methods: hierarchical and non-hierarchical methods. One of the clustering approaches could be selected after analysis. In other words, the desired number of clusters, *k*, is specified in advance, and each of the cases is assigned to one of the *k*-clusters to minimize the variance of the clustering of the internal techniques. In the non-hierarchical approach, for creating good communities, *k* is defined in advance so that the measurement items are based on the homogeneity of the communities. They are not nested clusters; hierarchical clustering is used to divide the samples.

### EM clustering

The concept of the EM algorithm stems from the Gaussian mixture model (GMM). The GMM method is one way to improve the density of a given set of sample data modelled as a function of the probability density of a single-density estimation method with multiple Gaussian probability density function to model the distribution of the data. In general, to obtain the estimated parameters of each Gaussian blend component if given a sample data set of the log-likelihood of the data, the maximum is determined by the EM algorithm to estimate the optimal model. Principally, the EM clustering method uses the following algorithm:

Input: Cluster number *k*, a database, stopping tolerance.

Output: A set of *k*-clusters with weight that maximize log-likelihood function.

1. Expectation step: For each database record *x*, compute the membership probability of *x* in each cluster *h* = 1,…, *k*.
2. Maximization step: Update mixture model parameter (probability weight).
3. Stopping criteria: If stopping criteria are satisfied stop, else set *j* = *j* +1 and go to (1).

In the analytical methods available to achieve probability distribution parameters, in all probability the value of the variable is given. The iterative EM algorithm uses a random variable and, eventually, is a general method to find the optimal parameters of the hidden distribution function from the given data, when the data are incomplete or has missing values.[5,6]

## Results and discussion

Clustering is an important means of data mining. Different algorithms can be used to separate data of a similar nature. Unlike the classification algorithm, clustering belongs to the group of unsupervised algorithms. Two representative clustering algorithms that are widely used are *K*-means and EM. Linear regression analysis was extended to the category-type dependent variable. Logistic regression, by using a linear combination of independent variables, is a statistical technique used to predict the possibility of occurrence of an event, i.e. its probability.[7] However, if a set of data are classified using logistic regression analysis only, it is not possible to guarantee the accuracy of the results.[7] In this paper, we use logistic classification after clustering the experimental data with the *K*-means and EM algorithm to solve the difficult problem of ensuring accuracy of the obtained results.

**Figure 1.**
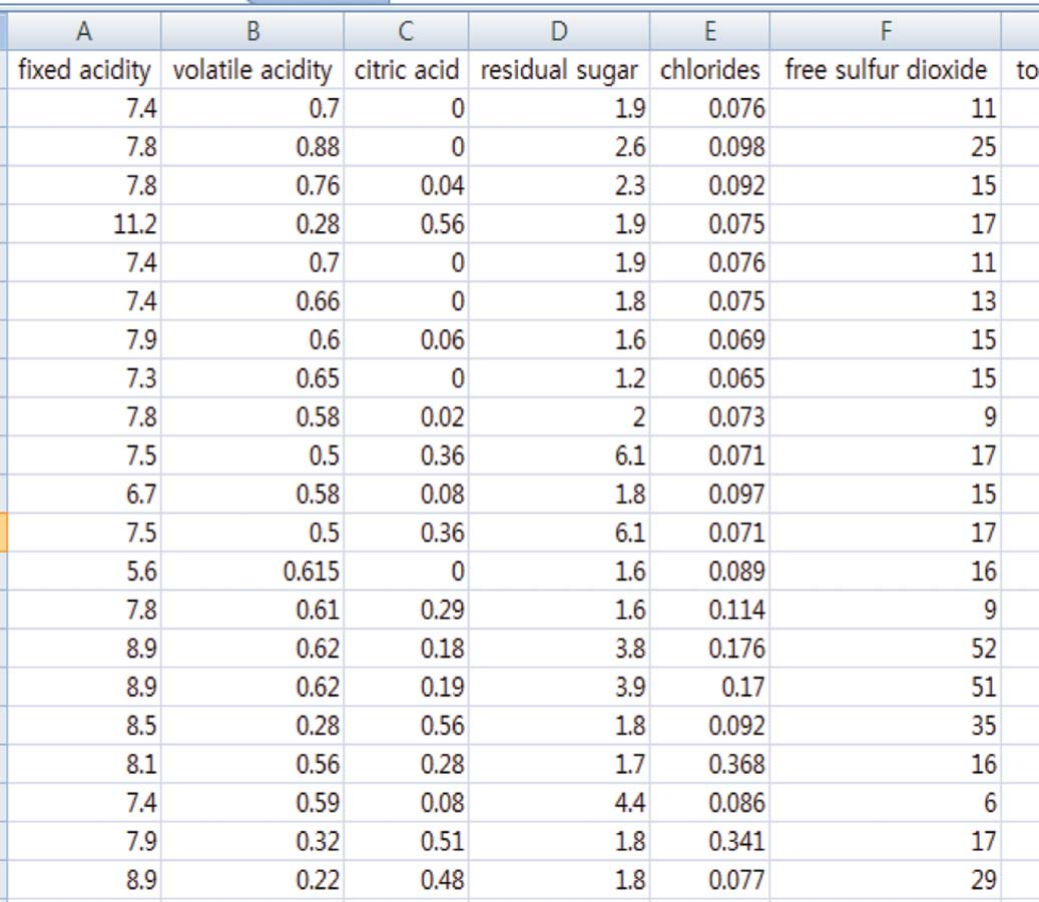
Part of experimental data.

EM clusters and *K*-means were applied for quality assessment of red wine by using Waikato Environment for Knowledge Analysis (WEKA) [8] and the performance of the two algorithms is compared based on logistic classification using the data set. Experimental data for quality evaluation of red wine are composed by attributes of a total of 13 pieces (Figure 1). Each attribute is defined in Table 1.

**Table 1.** Attributes of experimental data.

No.	Attribute	Type	Range
1	Colour	Binomial	0: red, 1: white
2	Fixed acidity	Numeric	[3.80, 15.90]
3	Volatile acidity	Numeric	[0.08, 1.58]
4	Citric acid	Numeric	[0.00, 1.66]
5	Residual sugar	Numeric	[0.60, 65.80]
6	Chlorides	Numeric	[0.01, 0.61]
7	Free sulphur dioxide	Numeric	[1.0, 289.00]
8	Total sulphur dioxide	Numeric	[6.00, 444.00]
9	Density	Numeric	[0.99, 1.04]
10	pH	Numeric	[2.72, 4.01]
11	Sulphates	Numeric	[0.22, 2.00]
12	Alcohol	Numeric	[8.00, 14.90]
13	Quality	Nomial	[0]very bad, [10] excellent

**Figure 2.**
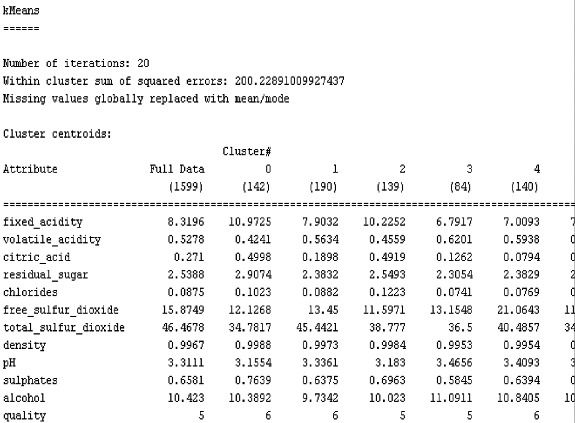
*K*-means experimental result.

**Figure 3.**
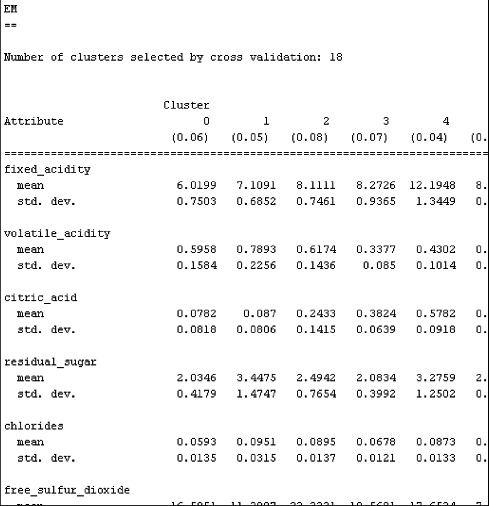
EM cluster experimental results.

It is possible to classify the red-wine quality data by using the logistic WEKA algorithm, which is provided to confirm the results shown in Figures 2 and 3. Among the 1599 total items of data, the number of data items classified correctly (956 items) indicates accuracy of 59.7874%, with the remaining 643 pieces (or 40.2126%) classified incorrectly. The results obtained by using the classification logistics WEKA provide low accuracy with high-processing speed, indicating that classification using only a logistic algorithm cannot guarantee the accuracy of the results. To compare the performance of the EM and *K*-means algorithms, wine-quality data were analysed ingredient content sugar and sour wine enemy of various compounds, the concentration and the alcohol concentration in the experimental clustering for all attributes. To compare the performance of *K*-means and EM, the same data are applied using the WEKA program.

Figure 2 shows the results obtained by *K*-means clustering in which the conditions were set as those for the EM clustering. The results showed that the processing speed was slower than that with the EM clustering, but the classification accuracy of the data was 94.7467% (Table 2), which is 7.3171% better than that obtained by EM. Naturally, the inaccuracy of the *K*-means was lower as compared to that of the EM algorithm. As a whole, further optimizations should be introduced to reduce the time.

**Table 2.** Experimental results applying *K*-means.

Instance classification	Percentage
Correctly classified instances	94.7467%
Incorrectly classified instances	5.2533%

Figure 3 shows the results obtained by clustering with the EM algorithm. The 13 attribute values for each attribute were clustered. When applied to the initial data, the logistic classification result of the clustering through the EM algorithm was confirmed to show more accurate classification results as compared to the classification without clustering. The accuracy was 59.7874% by analysing the data only using logistic classification (Table 3). When fitted into the logistic classification data, the accuracy of the data analysis was increased to 87.4296%, and the inaccuracy decreased to 12.5704%, respectively (Table 4).

**Table 3.** Experimental results applying EM.

Instance classification	Percentage
Correctly classified instances	59.7874%
Incorrectly classified instances	40.2126%

**Table 4.** Fitted logistic classification results using EM.

Instance classification	Percentage
Correctly classified instances	87.4296%
Incorrectly classified instances	12.5704%

## Conclusions

In this paper, logistic regression analysis was applied, and *K*-means and EM clustering methods were compared in terms of accuracy of the classification results and speed. The two methods were applied on data sets for evaluation of red-wine quality, such as sugar content and acidity, concentration of various compounds, alcohol concentration. Both of the clustering methods tested showed better accuracy than that achieved solely by classifying the experimental data with the logistic algorithm WEKA. The EM clustering method showed high accuracy (over 87%) of the results and high speed. The highest accuracy (over 94%) was achieved when the *K*-means algorithm was applied but it was more time-consuming than EM. To reduce the time, further optimizations should be carried out.
